# Laparoscopic colostomy takedown offers advantages over traditional surgery

**DOI:** 10.4103/0972-9941.28179

**Published:** 2006-12

**Authors:** Thomas M Schmelzer, William W Hope, David A Iannitti, Kent W Kercher, B Todd Heniford

**Affiliations:** Division of Gastrointestinal and Minimally Invasive Surgery, Carolinas Medical Center, Charlotte, NC, USA

It was a privilege to be able to review the article, “Laparoscopic reversal of Hartmann procedure” prior to its publication. The findings discussed are very similar to our early experience using the laparoscopic approach to reversing a colostomy.

The Hartmann procedure remains a standard operation for left-sided colon pathology that is not amenable to immediate reanastamosis. Reversing the colostomy to reestablish intestinal continuity is a major abdominal operation that historically results in extended recovery, prolonged hospital stays, significant rates of morbidity and mortality, and long-term complications such as hernias. Because of the risks associated with the operation, historically almost 50% of patients choose to forego colostomy reversal and keep their colostomy despite the physical and psychological challenges associated. We recently performed a study comparing our outcomes for 22 laparoscopic versus 22 open colostomy reversals and our results were comparable to those presented in this article. We demonstrated significantly less intraoperative blood loss, fewer postoperative complications, quicker return of bowel function and shorter hospital stays for the laparoscopic group. Based on these findings, we believe that a laparoscopic approach to colostomy reversal may increase the want or willingness of patients with a left-sided colostomy to undergo reanastomosis.

Our surgical technique for the laparoscopic reversal of a left-sided colostomy is similar to that discussed in the article with a few small differences. All patients receive a preoperative bowel preparation and an enema to evacuate the rectal stump. The patients are placed in the modified lithotomy position and a three-way Foley catheter is placed. Our port placement is different from that reported in this article. Port placement is related to the location of the prior abdominal incisions and the stoma. Either the colostomy site is used or an open cut down technique is performed to access the peritoneal cavity. If the prior midline incision extends to the epigastrium, the initial port is placed at the colostomy site. The colostomy is mobilized and the most distal segment of the colostomy is transected with a stapler at the mucocutaneous junction. The stapled colon is dropped back into the abdomen and a 10 mm balloon tipped trocar is placed in the prior colostomy site.

If a lower midline incision is present, initial access to the peritoneal cavity is typically gained with an open technique under direct vision in the left upper quadrant. A 5- or 10 mm port is placed in this incision. Typically, three ports are used in total with the third being located to the left of the midline superiorly. The benefit of these port placements is that the dense adhesions frequently found along the prior midline incision can be avoided and adhesiolysis minimized. Sharp adhesiolysis is performed to mobilize the splenic flexure and left colon. Excessive adhesiolysis of the prior midline incision is avoided. Next, the rectal stump is identified. If polypropylene sutures were placed on the rectum at the time of initial operation, they can greatly aid in the localization of the rectal stump. An additional aid in delineating the rectum is to insert a rectal dilator or rigid sigmoidoscope transanally. Once the rectal stump is visualized, it is dissected as needed to enable a stapled anastomosis. With extensive adhesions in the pelvis and in women who have had a prior hysterectomy, the bladder can be adherent to the rectum. This relationship can be difficult to interpret laparoscopically. At this point, 300-400 ml of saline is instilled through the three-way Foley catheter to ensure that the rectum is safely freed from it. The stapled left colon is then brought out through the prior ostomy site after the 10 mm balloon tip trocar is removed. The anvil for the circular stapler is secured within the left colon lumen. The colon is then returned to the abdomen and the balloon port is replaced. The circular stapled anastomosis is then performed under direct laparoscopic visualization.

Using this technique allows for certain advantages over an open technique and may explain the reduction in morbidity that we experienced in our study. As this article mentions, the laparoscopic approach gives better visualization of the splenic flexure allowing for routine mobilization. Mobilization of the splenic flexure in an open technique requires a larger midline incision, which can lead to an increased risk of postoperative morbidity. Splenic flexure mobilization is required often for this operation to reduce tension on the anastomosis with its resultant anastamotic dehiscence or stricture formation.

Avoiding the previous midline incision by accessing the abdomen laterally can help prevent the possibility of bowel injury. In our cases, the abdomen is always entered at a site remote from prior incisions. Keeping our ports on the left side of the body, the midline is often completely avoided. By doing this we have had no visceral injuries in our series.

There are additional long-term complications which we believe are reduced by using a laparoscopic approach. These include the formation of incisional hernias and, possibly, small bowel obstruction from additional adhesive disease. These have not been fully evaluated yet as longer follow-up periods are needed. The use of the laparoscopic technique for reversal of colostomies appears to offer distinct advantages over the open approach. It should be made clear, however, that this operation does require an experienced laparoscopic surgeon. Our conversion rate was 9%, which is similar to the rate reported in this article. Reasons for conversion include dense adhesions or inability to mobilize the rectal stump adequately; both of these can be determined with diagnostic laparoscopy and often does not require an extended period of time. Our experience and findings are in agreement with those put forth in this article. With less morbidity, shorter hospital stays and quicker recovery periods, laparoscopic colostomy closure should be attempted by surgeons with advanced laparoscopic skills.

